# Mesenchymal stem cells are resistant to carbon ion radiotherapy

**DOI:** 10.18632/oncotarget.2857

**Published:** 2014-12-03

**Authors:** Nils H. Nicolay, Yingying Liang, Ramon Lopez Perez, Tilman Bostel, Thuy Trinh, Sonevisay Sisombath, Klaus-Josef Weber, Anthony D. Ho, Jürgen Debus, Rainer Saffrich, Peter E. Huber

**Affiliations:** ^1^ Department of Radiation Oncology, Heidelberg University Hospital, Im Neuenheimer Feld, Heidelberg, Germany; ^2^ Heidelberg Institute for Radiation Oncology (HIRO), National Center for Radiation Research in Oncology, Im Neuenheimer Feld, Heidelberg, Germany; ^3^ Department of Molecular and Radiation Oncology, German Cancer Research Center (dkfz), Im Neuenheimer Feld, Heidelberg, Germany; ^4^ Department of Hematology and Oncology, Heidelberg University Hospital, Im Neuenheimer Feld, Heidelberg, Germany

**Keywords:** Mesenchymal stem cells, photon irradiation, carbon ion irradiation, radioresistance

## Abstract

Mesenchymal stem cells (MSCs) participate in regeneration of tissues damaged by ionizing radiation. However, radiation can damage MSCs themselves.

Here we show that cellular morphology, adhesion and migration abilities were not measurably altered by photon or carbon ion irradiation. The potential for differentiation was unaffected by either form of radiation, and established MSC surface markers were found to be stably expressed irrespective of radiation treatment. MSCs were able to efficiently repair DNA double strand breaks induced by both high-dose photon and carbon ion radiation. We have shown for the first time that MSCs are relatively resistant to therapeutic carbon ion radiotherapy. Additionally, this form of radiation did not markedly alter the defining stem cell properties or the expression of established surface markers in MSCs.

## INTRODUCTION

Mesenchymal stem cells (MSCs) were first characterized in bone marrow biopsies, but are also found in various other human tissues such as kidney, skin, vascular and adipose tissue, umbilical cord and placenta [1-3]. Unlike their hematopoietic counterparts, MSCs form a heterogeneous population that can be characterized by their fibroblast-like appearance and defining cellular functions such as their ability to adhere to plastic surfaces, proliferate in culture and differentiate along the osteogenic, adipogenic and chondrogenic lineage [4]. Although it would be advantageous to identify a unique MSC surface marker pattern in order to prospectively identify these cells, no broadly accepted marker set has been successfully established yet [5, 6].

MSCs have recently come into focus as a potential means of repairing tissue damage both by providing a supportive microenvironment and by differentiating into functional cells; several studies using preclinical data and animal models have shown an involvement of MSCs in the regeneration of damaged tissues such as myocardium, cartilage, lung, skin and neuronal tissues [7-9].

Radiotherapy as widely used for cancer therapy usually employs high-energy photons, but the use of particle-based treatments such as proton or carbon ion radiotherapy has become more prevalent in the treatment of tumors that are in close proximity to crucial organs at risk or exhibit a radioresistant phenotype [10]. Ionizing radiation (IR) exerts its effects by inducing DNA damage that can either be repaired by treated cells or otherwise results in cell cycle arrest, apoptosis or mutagenesis. Cellular radiation sensitivities vary considerably between tissues and have been linked to differences in the ability to effectively repair IR-induced damage [11].

MSCs have been shown to be relatively resistant to photon irradiation and preserve their defining functional characteristics after exposure to this form of IR [12, 13]. However, the influence of particle radiation as used in modern cancer treatment on the survival and functions of MSCs is largely unknown.

In this study, we examined radiation sensitivities of human MSCs to photon and carbon ion irradiation and investigated the influence of the different IR modalities on survival, mobility, differentiation potential and DNA repair capacities. Additionally, gene expression analyses were performed to assess potential gene regulation mechanisms influencing the response of MSCs to the different forms of radiation.

## RESULTS

### MSCs exhibit different sensitivities to photon and carbon ion irradiation

**Figure 1:**
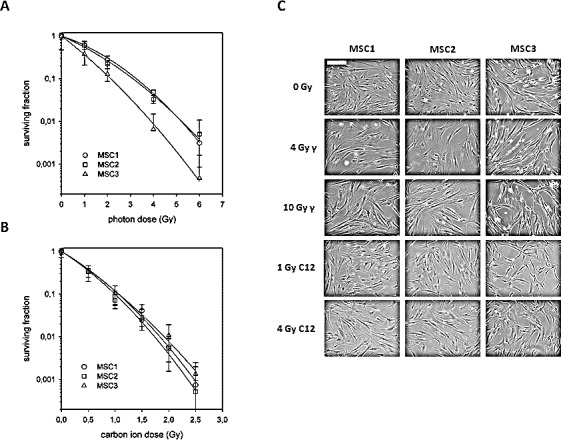
Mesenchymal stem cells exhibit different radiation sensitivities to photon and carbon ion irradiation (A) Clonogenic survival assays for three different MSCs after treatment with photon (upper panel) or carbon ion radiotherapy (lower panel). Error bars represent standard deviation. (B) Images of unstained MSCs showing no measurable difference in morphology after treatment with different doses of photon or carbon ion irradiation (20x objective, scale bar 100μm).

To assess the effects of photon and carbon ion irradiation on the survival of mesenchymal stem cells, clonogenic assays of bone marrow-derived MSCs from three different healthy donors were performed. In line with previous findings, all tested MSCs showed a relatively radioresistant phenotype after photon irradiation with small but non-significant differences regarding individual radiation sensitivities (Figure 1A). To assess the effects of particle irradiation on MSCs, cells were exposed to the extended Bragg peak of a carbon ion beam, and clonogenic survival was measured. All three tested MSCs showed a markedly decreased survival after carbon ion irradiation compared to the effects seen for photon treatment, translating into relative biological effectiveness values (endpoint 10 % survival, D0.1) of 3.08, 3.10 and 2.00 for MSC1, MSC2 and MSC3, respectively (Figure 1B).

### MSC morphology remains stable after particle irradiation

MSCs have a characteristic spindle-shaped appearance, similar to that of differentiated fibroblasts. At both 4 Gy and 10 Gy photon irradiation, MSC morphology remained largely unchanged as has been reported before [12]. Similarly, even high doses of carbon ion irradiation up to 4 Gy did not alter the cellular shape or size (Figure 1C). No morphological signs of increased apoptosis could be detected at 24 hours after photon or particle irradiation using light microscopy.

### MSC adhesion and migration are unaffected by photon and particle irradiation

**Figure 2:**
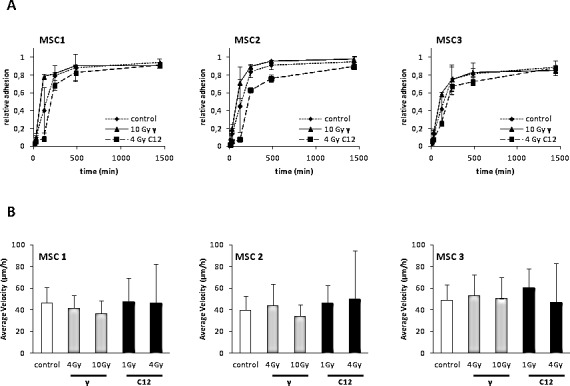
Photon or carbon ion irradiation do not impair the adhesion or migration abilities of MSCs (A) Relative adhesion rates of MSCs up to 24 hours after treatment with 10 Gy photon or 4 Gy carbon ion irradiation. (B) Average velocity of MSCs after irradiation as assessed by time-lapse microscopy. Error bars show standard deviation.

The ability of MSCs to adhere to plastic surfaces was measured up to 24 after treatment with 10 Gy photon or 4 Gy carbon ion irradiation (Figure 2A). Particle-irradiated MSCs were found to exhibit a short delay in the onset of adherence compared to photon- or mock-treated samples. However, the observed small difference in adherence at early time points was abolished at 24 hours after treatment, with highly similar adherence rates above 80% of MSCs for both radiation modalities. Additionally, we did not measure a significant difference between mock-treated and irradiated MSC samples at 24 hours after treatment, suggesting that the ability to adhere is not hampered even by high doses of photon or particle radiotherapy.

MSC migration was measured by time-lapse microscopy (supplementary Figure 1). Average velocity was comparable between all MSCs without treatment (Figure 2B). Treatment with 4 or 10 Gy photons or 1 or 4 Gy carbon ions did not reduce average velocity in any of the tested samples, showing that MSC motility was unaffected by high-dose photon or particle irradiation.

### Photon and particle irradiation do not affect the differentiation potential of MSCs

The ability for adipogenic or chondrogenic differentiation is a hallmark of MSCs. To investigate if this intrinsic differentiation potential was affected by carbon ion irradiation, cells were treated with a single radiation dose of 10 Gy photons or 4 Gy particles. Immunocytochemical studies were performed as described above to assess differentiation features.

**Figure 3:**
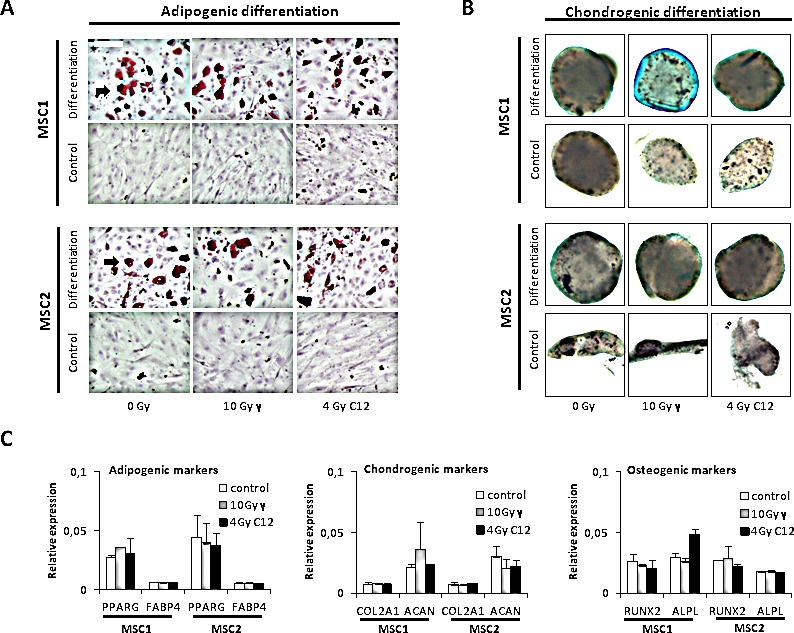
Photon or carbon ion irradiation do not impair the differentiation potential of MSCs (A) Oil red O staining for adipogenic differentiation in two MSC samples after irradiation with 10 Gy photon or 4 Gy carbon ion irradiation. Pictures were taken at 100x magnification (B) Alcian blue staining of MSC spheroids after treatment, 40x magnification. (C) Relative expression levels of adipogenic, chondrogenic and osteogenic differentiation markers after radiotherapy. Error bars represent standard deviation.

Neither photon irradiation nor high-dose carbon ion treatment affected the ability of MSCs to form lipid inclusions as assessed by oil red O staining, suggesting that the adipogenic differentiation potential was intact (Figure 3A). Similarly, Alcian blue staining revealed intact chondrogenic differentiation after high-dose photon or particle irradiation and culturing of MSCs in differentiation media (Figure 3B). Gene array analyses demonstrated stable but low expression of established differentiation markers, showing on the transcriptional level that irradiation did not abrogate the cells' differentiation potential and did not induce differentiation as reported for other cells of mesenchymal origin [14] (Figure 3C). Taken together, these data indicate that the ability of MSCs to differentiate is unaffected by irradiation at the tested doses irrespective of the radiation modality.

### MSC surface markers are stably expressed independent of the radiation treatment

**Figure 4:**
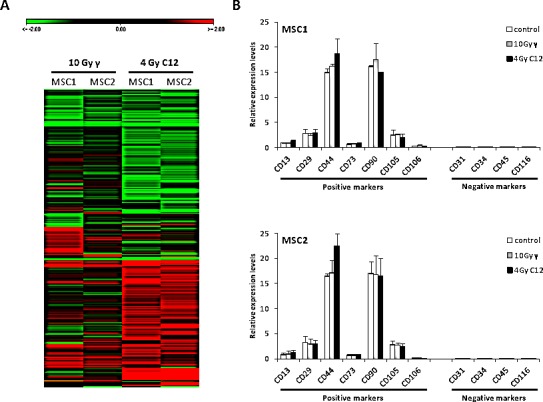
The expression of MSC surface marker genes is not influenced by photon or carbon ion irradiation (A) Heat map showing similar patterns of downregulation (green panel) or upregulation (red panel) in MSCs after irradiation with 10 Gy photons or 4 Gy carbon ions. (B) Relative expression levels of positive and negative MSC surface marker genes after irradiation. Error bars represent standard deviation.

Gene expression patterns of MSC1 and MSC2 were assessed after treatment of cells with high-dose photon or carbon ion radiotherapy, using a whole human genome microarray. Patterns of mRNA regulation were found to be very similar between photon and particle-irradiated samples in the two tested MSCs, suggesting a similar response to both types of radiation (Figure 4A). Expression of established mesenchymal stem cell markers was measured in MSCs at 6 hours after mock-treatment or irradiation with 10 Gy photons or 4 Gy carbon ions. High levels of positive surface markers CD13, CD29, CD44, CD73, CD90, CD105 and CD106/VCAM-1 were detected in both tested MSCs, and the hematopoietic negative markers CD31 CD34, CD45 and CD116 were not expressed to detectable levels (Figure 4B). After treatment with 10 Gy photons or 4 Gy carbon ions, expression levels did only change marginally for any of the assessed surface markers, showing that all established MSC surface markers were robustly expressed, and even high doses of photon or particle irradiation did not change the molecular profile of the tested MSCs.

### Photon and carbon ion irradiation do not increase apoptosis levels in MSCs

**Figure 5:**
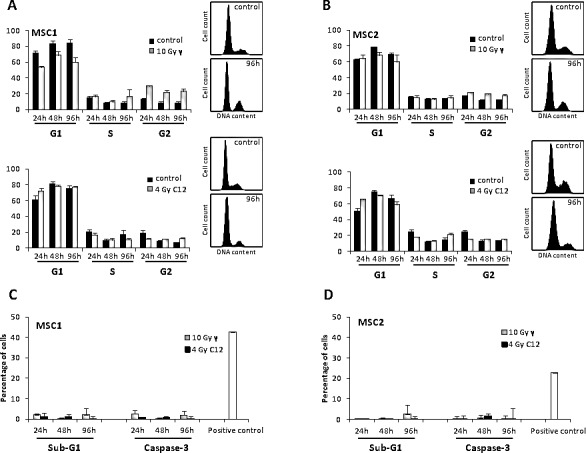
Photon and carbon ion irradiation of MSCs leads to G2 phase arrest but no increase in apoptosis Cell cycle distribution of MSC1 (A) and MSC2 (B) cells after treatment with 10 Gy photon radiation (upper panels) or 4 Gy carbon ion radiation (lower panels). (C, D) Percentage of apoptotic MSC1 and MSC2 cells after 10 Gy photon or 4 Gy carbon ion irradiation as assessed by sub-G1 population and caspase-3 activation. Error bars show standard deviation.

Cell cycle profiles of MSCs were analyzed by FACS after photon and carbon ion irradiation. IR treatment with 10 Gy photons resulted in a small increase of G2 phase cells as described previously [12] (Figure 5A, B). This effect appeared to be less pronounced after carbon ion irradiation and occurred only during later time points between 48 and 96 hours. The percentage of S phase cells was found to stably range between 12 and 20 %, suggesting continuous cellular proliferation even after high doses of photon or carbon ion irradiation.

Measurements of cellular sub-G1 population and caspase 3 activation were used as read-outs for apoptosis induced in MSCs by photon and carbon ion radiation. MSC samples showed no significant increase in the percentage of sub-G1 cells after high-dose treatment with photon or particle radiation compared to the untreated controls (Figure 5 B, C). Similarly, caspase-3 activation was found to be comparable in irradiated MSCs and untreated control samples both for photon and carbon ion treatment, suggesting no increase in apoptosis after either form of radiation.

### MSCs efficiently repair DSBs caused by photon and carbon ion irradiation

**Figure 6:**
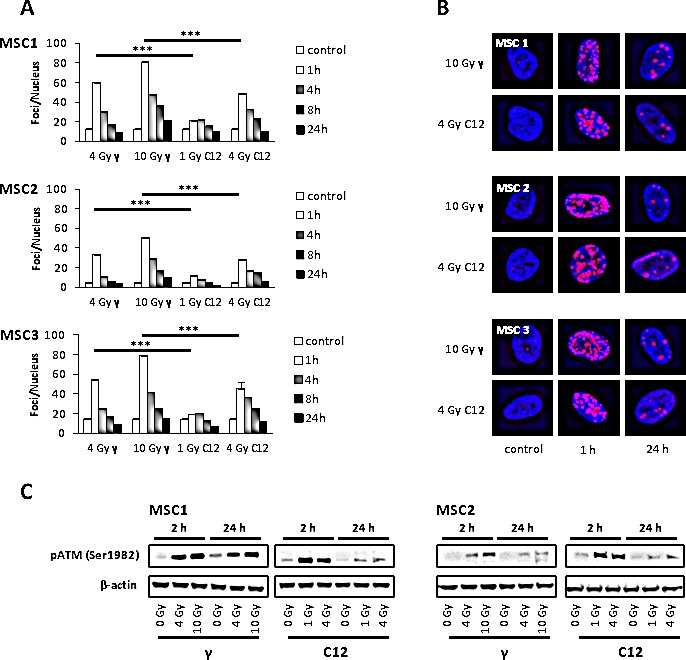
MSCs efficiently repair DNA double strand breaks induced by photon or carbon ion radiotherapy (A) Number of γH2AX foci in MSCs at various time points after irradiation with different photon and carbon ion doses as measured by immunostaining. *** *P*<0.001 (B) Sample pictures of γH2AX foci at high photon and carbon doses (400x magnification). (C) Western blot analyses of phosphorylated ATM protein at 2 and 24 hours after irradiation with photons or carbon ions.

To investigate the ability of irradiated MSCs to efficiently repair potentially lethal DNA damage, immunofluorescence analyses of γH2AX foci as markers of DNA double strand breaks were performed. Photon irradiation with 4 Gy led to a 3 to 4-fold increase in the amount of γH2AX foci in all MSC samples at 1 hour after treatment, with a quick reduction in the foci numbers at 4 and 8 hours and a return to baseline levels at 24 hours (Figure 6A). Higher photon doses of 10 Gy increased the initial foci numbers even further, but at 24 hours after irradiation, the vast majority of DNA double strand breaks as measured by γH2AX foci were found to be repaired.

Carbon ion doses of 1 and 4 Gy resulted in significantly lower numbers of foci per cell in all tested MSC samples at 1 hour after treatment when compared to the biologically equivalent photon doses of 4 and 10 Gy (*P*<0.001 for all tested samples, Student's t-test); however, foci of particle-irradiated MSCs appeared bigger than those of photon-treated cells (Figure 6B). Cellular foci numbers in all tested MSCs were found to reach baseline levels at 24 hours after treatment with carbon ion radiation, suggesting efficient repair of DNA double strand breaks even after high doses of particle irradiation.

Western Blot analyses showed strong phosphorylation of ataxia teleangiectasia-mutated (ATM) protein at 2 hours both after photon and particle irradiation and only residual pATM signals after 24 hours (Figure 6C), corresponding to the low levels of residual γH2AX foci at that time point.

## DISCUSSION

This analysis compared the effects of photon and carbon ion irradiation on the survival and functional properties of human mesenchymal stem cells. While previous analyses have shown relative radioresistance of MSCs after photon irradiation, no data are available regarding other forms of radiation such as carbon particle radiotherapy despite their clinical use [12, 13, 15].

In comparison to photon radiation, carbon ion radiation deposits its energy more densely, resulting in more clustered DNA damage and DNA double-strand breaks; therefore, higher relative biological effectiveness (RBE) values have been reported, ranging between 1.4 and 4.8 depending on the biological model, endpoint and beam energy [16, 17]. The RBE values of the tested human MSC samples in this dataset were found to be between 2.0 and 3.1 with respect to the survival fraction 10% endpoint (D0.1). Radiosensitivity is influenced by various factors and depends on the cellular ability to deal with the potentially lethal DNA damage caused by IR, especially double-strand breaks [18]. MSCs were able to efficiently repair these critical DNA lesions both after photon and after carbon ion irradiation as assessed by γH2AX immunostaining. While these cells exhibited less γH2AX foci directly after exposure to particle radiation than after photon treatment, carbon ion-induced foci appeared bigger; this finding corresponds to previously published data suggesting larger foci sizes along carbon ion tracks due to the increased creation of clustered DNA damage by densely ionizing particle radiation [19]. The ability of MSCs to swiftly repair both photon and particle-induced DNA double strand breaks was also reflected by the activation of the ataxia teleangiectasia-mutated (ATM)-dependent DNA damage signaling pathway. Phosphorylation of ATM is an early step in the DNA damage-signaling cascade [20]. We found that ATM phosphorylation as initially observed in MSCs after IR was resolved or strongly reduced after 24 hours both for photon and carbon ion-irradiated samples, suggesting efficient repair of the vast majority of DNA damage irrespective of the radiation modality. The importance of the ATM pathway for the radiation response of MSCs has been highlighted by previous publications, and the upregulation of different DNA damage recognition and repair proteins has been correlated with increased radiation resistance in these cells [21-23]. Similar results have been obtained for cancer stem cells such as glioma or breast cancer stem cells, where an upregulation of the DNA damage repair capacity has been linked to their reported high radioresistance that may be responsible for tumor recurrence [24-26].

As published for other cell types, both photon and carbon ion irradiation resulted in a small increase of G2 phase cells [27, 28]. MSCs in S phase stably remained between 10 and 20% even after high doses of photon or particle treatment, suggesting continuous proliferation and corresponding well with a relatively slow cell doubling time of around 40 hours. Several papers have demonstrated a connection between a prolonged doubling time and a relative increase in radioresistance, as cells may have more time to efficiently deal with IR-induced DNA damage [29, 30]. Additionally, we observed no increase in apoptosis levels even after high doses of photon or carbon ion treatment, supporting the concept of relatively high radioresistance.

As no general pattern of cellular surface markers has been established, MSCs are commonly identified by their functional characteristics, e.g. their ability to proliferate *in vitro*, adhere to plastic surfaces and differentiate upon induction [4, 6]. We have previously shown that these MSC characteristics are largely preserved after high-dose photon irradiation [12], but the influence of other forms of IR, especially clinically used particle radiotherapy, on MSCs remains unknown. The data presented here demonstrated that MSC morphology remained widely unchanged, and the cells kept their ability to expand *in vitro* after exposure to high-dose photon or carbon ion irradiation. Additionally, the cells' adherence potential was not significantly altered after both forms of radiation treatment. Adhesion to plastic surfaces is commonly regarded as one of the defining functional MSC characteristics and is used to select those cells in culture. Previous publications have described an unaffected adhesion potential after photon irradiation, and these findings have been backed by gene expression analyses showing an upregulation of various genes involved in cellular adhesion after irradiation of MSCs [12, 31].

Similarly, the ability to differentiate along the adipogenic or chondrogenic lineage constitutes a key feature of MSCs, and this ability has been linked to the regenerative potential of these cells as demonstrated *in vitro* and *in vivo* [32]. We found that all analyzed MSC samples were able to differentiate after high-dose photon and carbon ion irradiation. Previously, Li et al. reported a dose-dependent reduction of the differentiation potential of MSCs after IR treatment, however, in their analysis the differentiation potential was still maintained after high doses [33]. An analysis using ^56^Fe ion radiotherapy showed that after low doses up to 1 Gy, MSCs were still able to undergo osteogenic differentiation [34]. In our dataset, even considerably higher particle doses up to 4 Gy did not abolish the MSCs' ability to differentiate. The expression of various differentiation markers remained largely unchanged after high doses of carbon ion radiation, suggesting that on the transcriptional level, the ability for differentiation was not affected. Additionally, our data showed that neither photon nor particle radiation induced MSC differentiation, as has been reported for irradiated fibroblasts [14, 35].

Gene array data showed high expression of established positive MSC surface markers and no measurable levels of the negative, hematopoietic markers; expression of both marker sets was not affected by either photon or carbon ion irradiation, and both tested MSC samples showed comparable expression patterns for their surface markers. These findings suggest that MSCs did not change their established molecular signature upon photon or particle irradiation [4, 36], at least not in the context of an immediate radiation response up to 6 h.

The relative resistance of MSCs to particle irradiation and the preservation of their defining stem cell traits is a prerequisite for a potential regenerative function of these cells after radiation-induced tissue damage. As carbon ion radiotherapy is often used for the treatment of cancers of the skull base and head/neck, treatment commonly affects various organs at risk, e.g. the temporomandibular joints or the salivary glands [37]. Additionally, due to their physical properties, carbon ion radiotherapy has been established as a useful modality for re-irradiation after previous radiation treatment. In this context, the additional applied dose puts the patients at an increased risk of treatment-induced severe side effects. As shown in animal models for photon radiotherapy, MSC-based treatments may eventually also become a useful and powerful means for attenuating the often problematic side effects caused by particle radiotherapy [38, 39].

Similar to their role in the bone marrow microenvironment, MSCs have been described as an integral part of the tumor stroma [40-42]; and the secretion of cytokines and growth factors by the tumor tissue was linked to the recruitment of MSCs to the tumor site [43, 44]. MSCs in turn have been shown to create an advantageous tumor microenvironment by the secretion of paracrine factors such as CCL5, thereby promoting tumor growth and metastasis [41, 45]. The radioresistant phenotype of MSCs as observed after both photon and carbon ion irradiation may lead to an increased survival of these cells after tumor radiotherapy. Additionally, the reported secretion of cytokines like TGF-β (transforming growth factor β), GM-CSF (granulocyte/macrophage-colony stimulating factor) or TNF-α (tumor necrosis factor α) by MSCs after exposure to ionizing radiation may even have a protective effect on the irradiated tumor cells [46, 47]. Therefore, the relative radiation resistance of MSCs may enhance tumor survival after radiotherapy and even promote further tumor development. Therefore, further research is needed to elucidate the clinical implications of the radiobiological properties of MSCs as reported here.

Taken together, our data show that bone marrow-derived MSCs were relatively resistant to both photon and carbon ion radiation. Additionally, it was shown for the first time that the defining stem cell characteristics of MSCs are preserved even after high doses of particle radiotherapy.

## METHODS

### Cells and cultures

Primary human MSC1, MSC2 and MSC3 mesenchymal stem cells were sampled from bone marrow biopsies of three healthy voluntary donors and isolated as previously published [48]. Cells were proliferated in Mesenchymal Stem Cell Growth Medium (*MSCGM^TM^*, Lonza, Basel, Switzerland), supplemented with *MSCGM^TM^* SingleQuots (Lonza) and were kept in a humidified incubator at 37°C and 5% CO_2_. Written consent from donors was obtained prior to the harvesting procedure according to current ethics guidelines, and this study was approved by the Independent Ethics Committee of the University of Heidelberg Medical Faculty.

### Clonogenic survival assays

Cells were plated and allowed to attach for 6 hours before irradiation. Photon radiation was applied using a 6 MeV linear accelerator (dose rate of 3 Gy/min); carbon ion radiotherapy was performed by an extended Bragg peak (specific energy = 128 ± 7 MeV/u; linear energy transfer = 91.5 ± 1.5 keV/μm) at the Heidelberg Ion Therapy Center. All radiation experiments were carried out at room temperature. After treatment, cells were grown for 14 days to allow for colony formation. Colonies were fixed with 25% acetic acid (v/v) in methanol and stained with crystal violet solution. Colonies that contained more than 50 cells were then counted on a light microscope. All clonogenic assays were performed in triplicate. The surviving fraction was calculated according to the following formula: (#colonies/plated cells)_treated_/(#colonies/#plated cells)_untreated_. Relative biological effectiveness values for carbon ion irradiation were calculated by the following formula: (photon dose_10% survival_)/(carbon ion dose_10%survival_).

### Adhesion measurements

Immediately after photon or carbon ion irradiation, 100 MSCs were plated in each well of a 24-well plate. Attached cells were counted at various time points after plating using a light microscope, and the attachment efficiency was calculated as the ratio between attached and plated cells. All measurements were performed in triplicate.

### Migration measurements

Migratory behavior of MSCs after irradiation was measured by time-lapse microscopy on an IX70 inverted microscope (Olympus, Hamburg, Germany) equipped with an incubator box. Cells were grown in 24-well plates and imaged every 10 minutes over a period of 40 hours. Acquired time-lapse data were quantified by manual single-cell tracking using ImageJ software (National Institutes of Health, Bethesda, USA). Experiments were performed in triplicate; tracks of at least 10 cells from three positions in each well were analyzed for each condition.

### Cell cycle analyses

For the analysis of cell cycle profiles, cells were harvested and washed before fixation using ice-cold 70% ethanol. Cells were then centrifuged and incubated with 10μg/mL propidium iodide solution containing 200μg/mL RNase A. A LSR II system (Becton-Dickinson, Heidelberg, Germany) was used for fluorescence-assisted cell sorting. For each experiment, 10 000 events were counted, and cell cycle profiles were modeled using the FlowJo 7.6.5 software (FlowJo LLC, Ashland, USA).

### Apoptosis measurements

After treatment, cells were harvested and fixed in 4% paraformaldehyde solution before resuspension in ice-cold 70% ethanol. Cells were then washed thrice in PBS containing 200μg/mL RNase A and 5g/L bovine serum albumin. Cells were centrifuged and incubated with the caspase 3 antibody (1:20, BD Pharmingen, Heidelberg, Germany) for 1 hour at room temperature. Analyses were performed on a LSR II analyzer system. 10 000 events were recorded for each treatment condition. Hypoxic MSCs were used as positive controls for the apoptosis measurements.

### Cellular differentiation experiments

MSCs in log phase were plated in 24-well plates and irradiated with 4 and 10 Gy photons or 1 and 4 Gy carbon ions. After 24 hours, medium was replaced by differentiation media, and cells were grown for 14 days.

Adipogenic differentiation was induced with Dulbecco's Modified Eagle Medium containing 10% fetal calf serum, 2mM L-glutamin, 1μM dexamethasone, 500μM 1-methyl-3-isobutylxanthine, 10μg/mL insulin and 100 U/mL penicillin/streptomycin. Differentiation media were exchanged twice weekly. Adipocytic differentiation was shown by oil red O staining. In brief, after washing and fixation with 4% paraformaldehyde, cells were treated with 60% isopropanol for 5 min before staining with oil red dissolved in 60% isopropanol. Cells were exposed to hematoxylin for 2 min and covered in glycerol. For chondrogenic differentiation, the STEMPRO® Chondrogenesis Differentiation Kit (Gibco Life Technologies, Frankfurt, Germany) was used according to the manufacturer's instructions. In brief, cells were grown either in differentiation medium or in standard culturing medium for control experiments. After 2 weeks, cells were fixed with 4% paraformaldehyde and washed with distilled water. Cells were stained with Alcian Blue dissolved in 60% ethanol and 40% acetic acid overnight at room temperature and afterwards washed in de-staining solution (60% ethanol, 40% acetic acid). Pictures were taken on a light microscope at 100x magnification for adipogenic and 40x for chondrogenic specimens.

### Gene expression analysis

Gene expression effects of photon and carbon ion irradiation on MSCs were assessed using a whole human genome microarray 4x44k (Agilent Technologies, Böblingen, Germany). Log phase cells were irradiated with 10 Gy photons or 4 Gy carbon ions or mock-irradiated, and RNA was extracted at 6 hours after treatment using an RNeasy Mini Kit (Qiagen, Hilden, Germany). Data were extracted with the Agilent feature extraction software (Agilent version 9.1) and analyzed. Statistical analysis was performed using paired Student's t-test.

### DNA repair foci

MSCs were plated on coverslips and allowed to attach before irradiation with photons (4 and 10 Gy) or carbon ions (1 and 4 Gy). Cells were fixed with 4% paraformaldehyde at different time points after treatment as indicated in the Results section. Cells were permeabilized with 0.3% Triton X-100 in PBS, and unspecific binding was blocked with 3% bovine serum albumin in PBS. Cells were incubated with a mouse monoclonal antibody against γH2AX (Biolegend, London, UK) overnight at 4°C, and with the secondary anti-mouse antibody coupled with Cy3 (Jackson ImmunoResearch, Newmarket, UK) for 1 hour at room temperature. Nuclei were labeled with 4′6-diamidino-2-phenylindole, and images were taken with 40x magnification on an Axioplan2 microscope (Zeiss, Jena, Germany) and foci automatically counted using Metafer software (Metasystems, Altlussheim, Germany). Experiments were performed at least three times. Statistical analysis was performed using the two-sided Student's t-test.

### Western Blotting

MSCs were irradiated with photons at 4 and 10 Gy and carbon ions at 1 and 4 Gy before harvesting at 2 and 24 hours after treatment. Each sample containing 10μg of total protein from whole-cell lysates was run on a polyacrylamide gel and transferred to a polyvinylidene difluoride membrane (Millipore, Darmstadt, Germany). Membranes were probed with antibodies against phospho-ATM and β-actin (Cell Signaling Technology, Leiden, Netherlands). Blots were visualized on X-ray film using a horseradish-peroxidase kit (Cell Signaling Technology).

## SUPPLEMENTARY MATERIAL, FIGURES


