# Supplementary material for: Mesenchymal stem cells are resistant to carbon ion radiotherapy
Source: Oncotarget. 2014 Dec 3;6(4):2076–87. doi: 10.18632/oncotarget.2857 (PMC4385837; doi:10.18632/oncotarget.2857)
Supplement: Supplementary file 1 [file oncotarget-06-2076-s001.pdf]

Mesenchymal stem cells are resistant to carbon ion radiotherapy

Supplementary Material

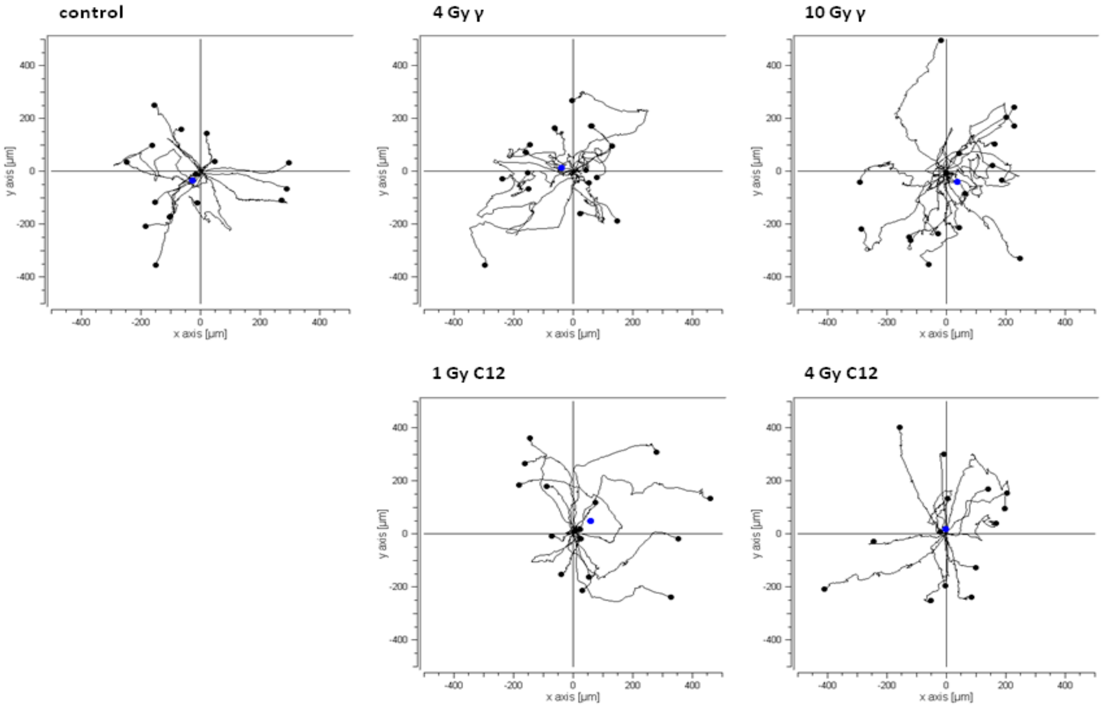

Supplementary figure 1: MSC migration is not impaired by photon or carbon ion irradiation. Tracks of MSC1 cells after photon or carbon ion irradiation as extracted from time-lapse microscopy images over a time period of 24 hours.
